# Overtime Work as a Predictor of Major Depressive Episode: A 5-Year Follow-Up of the Whitehall II Study

**DOI:** 10.1371/journal.pone.0030719

**Published:** 2012-01-25

**Authors:** Marianna Virtanen, Stephen A. Stansfeld, Rebecca Fuhrer, Jane E. Ferrie, Mika Kivimäki

**Affiliations:** 1 Finnish Institute of Occupational Health, Helsinki, Finland; 2 Wolfson Institute of Preventive Medicine, Queen Mary University of London, London, United Kingdom; 3 Department of Epidemiology, Biostatistics and Occupational Health, McGill University, Montreal, Canada; 4 Department of Epidemiology and Public Health, University College London, London, United Kingdom; 5 School of Community and Social Medicine, University of Bristol, Bristol, United Kingdom; Catholic University of Sacred Heart of Rome, Italy

## Abstract

**Background:**

The association between overtime work and depression is still unclear. This study examined the association between overtime work and the onset of a major depressive episode (MDE).

**Methodology/Principal Findings:**

Prospective cohort study with a baseline examination of working hours, psychological morbidity (an indicator of baseline depression) and depression risk factors in 1991–1993 and a follow-up of major depressive episode in 1997–1999 (mean follow-up 5.8 years) among British civil servants (the Whitehall II study; 1626 men, 497 women, mean age 47 years at baseline). Onset of 12-month MDE was assessed by the Composite International Diagnostic Interview (CIDI) at follow-up. In prospective analysis of participants with no psychological morbidity at baseline, the odds ratio for a subsequent major depressive episode was 2.43 (95% confidence interval 1.11 to 5.30) times higher for those working 11+ hours a day compared to employees working 7–8 hours a day, when adjusted for socio-demographic factors at baseline. Further adjustment for chronic physical disease, smoking, alcohol use, job strain and work-related social support had little effect on this association (odds ratio 2.52; 95% confidence interval 1.12 to 5.65).

**Conclusions/Significance:**

Data from middle-aged civil servants suggest that working long hours of overtime may predispose to major depressive episodes.

## Introduction

Common mental disorders, such as depression, are an important public health concern [1], [2]. According to projections by the World Health Organisation, depressive disorders will be the leading cause of disease burden in high-income countries by 2030 [1]. In addition to human misery, mental disorders often result in substantial work impairment and lost work days [2], [3]–[5].

Recent prospective studies, although not entirely consistent [6]–[11] suggest that long working hours may increase the risk of various adverse outcomes, including psychological distress and symptoms of depression and anxiety [8], [9], decline in cognitive function [10], and new-onset sleep disturbances [11]. However, to date, little is known about the relationship between long working hours and clinical depression. In a follow-up of Canadians over 1–2 years [12], working 41 hours or more per week compared to 35–40 hours was associated with a higher rate of new-onset major depressive episode (MDE) among women but not among men. Another study with a 24-year follow-up did not find any association between “overtime work” and incidence of depressive disorder which included also cases of subclinical depression [13]. A Japanese study that used employee insurance company records found no association between long working hours and mental disorders [14]. Several factors in previous studies may have contributed to the mixed evidence, including the dichotomisation of working hours, use of retrospective reports of hours worked, and clinically non-validated disease outcome measures, as well as extensive follow-up periods potentially introducing effect dilution bias.

In this study, we examined the association between overtime work and the onset of a major depressive episode in a sample of British civil servants who were free from psychological morbidity at baseline. We used the Composite International Diagnostic Interview (CIDI) [15], [16] to assess the onset of 12-month DSM-III-R-defined MDE [17], as this instrument is considered as the gold standard for large epidemiologic studies.

## Methods

### Ethics statement

Informed consent was obtained from all participants and the University College London Medical School Committee on the Ethics of Human Research approved the protocol.

### Participants and Procedure

Recruitment to the Whitehall II study (phase 1) took place between late 1985 and early 1988 among all office staff, aged 35 to 55 years, from 20 London-based Civil Service departments [18]. The response rate was 73% (6895 men and 3413 women).

The data for exposure and outcome measures for the present study are drawn from two survey phases; phase 3 (1991–3), the baseline for the present analyses when working hours were measured for the first time; and phase 5 (1997–99), follow-up, when DSM-III-R MDE was evaluated for the first and only time. The number of participants who worked full time (≥7 hours per day) at phase 3 baseline was 7287 and a further 7024 (96%) had complete data on covariates at baseline, 6251 responded at follow-up, and 4366 were still employed at follow-up. We selected those who were employed to reduce misclassification of working hours during the follow-up. In this sub-sample, 2724 had also completed the CIDI-interview at follow-up. Finally, we excluded 601 participants with psychiatric morbidity at baseline defined as caseness on the 30-item General Health Questionnaire (GHQ-30 total score ≥5) [19]. In relation to diagnosed mental disorders, especially mood and anxiety disorders, the GHQ has shown good clinical validity as a screening instrument [19]–[21]. The GHQ-30 has been validated specifically against the Clinical Interview Schedule in Whitehall II data, giving a cut-off point 4/5 for dividing ‘non-cases’ from ‘cases’ [21]. The final analytic sample consisted of 2123 employees followed for a mean of 5.8 (S.D. = 0.4, range 3.8 to 7.2) years.

Presence of an MDE in the preceding 12 months was ascertained during the clinical health examination using the University of Michigan version of the Composite International Diagnostic Interview (UM-CIDI) adapted for self-administered computerised interview [15], [16]. The program used operationalized criteria for diagnoses in the Diagnostic and Statistical Manual of Mental Disorders (DSM-III-R) [17]. In addition to the criteria for the presence and duration of the DSM-III-R symptoms, the definition of a MDE required that the episode also fulfilled criteria for impairment and change in function, and was not due to organic conditions, bereavement, or mania. The CIDI interview was commenced after the beginning of screening at phase 5: all participants attending the screening clinic after the CIDI was introduced were invited to complete the interview.

We determined working hours at baseline with the following question: “On an average weekday, approximately how many hours do you spend on the following activities (if applicable): Work (daytime and work brought home)?” Response categories covered working hours from 1 hour to 12 hours. As previously [22], we divided the participants into the following groups: 7–8 working hours/day (the Civil service standard, i.e. no overtime work); 9 hours/day (1 hour of overtime work a day); 10 hours/day (2 hours of overtime work); 11–12 hours/day (3–4 hours of overtime work).

Data on socio-demographic factors, health-related behaviours, and physical health at baseline were used as covariates in the analyses. Socio-demographic factors included sex, age, marital status (married/cohabiting vs not), and socioeconomic position, SES (as indicated by occupational grade grouped into six levels). Alcohol consumption was classified as 0, >0 to 14 units/week for women and 0, >0 to 21 units/week for men (alcohol consumption within the recommended limits); and >14 units for women and >21 units for men (alcohol consumption over the recommended limits) [23]. Smoking was assessed by questions on whether the respondent was a current, past, or never-smoker. Poor physical health was indicated by the presence of at least one of the following conditions: report of longstanding illness, disease, or medical condition for which the participant had sought treatment in the 12 months before the baseline survey; presence of coronary heart disease (for details [24]). Work stress was operationalised as self-reported job demands, job control, and social support at work [25], [26]. As an indicator of job strain we formulated a variable in which the demand and control scales were dichotomized based on their median scores, and participants were assigned to 1 of 4 categories according to scores on each dimension: low strain (low demand and high control), passive (low demand and low control), active (high demand and high control), or high strain (high demand and low control) – this variable corresponds the original definition of job strain by Karasek [25]. Social support was low when the respondent was in the lowest third of work social support. Employment status at follow-up (employed vs not) was derived from the follow-up survey.

### Statistical analysis

Descriptive statistics were carried out using χ^2^ and general linear modeling to examine heterogeneity across study variables. The association between working hours and new-onset MDE was examined with binary logistic regression analysis sequentially adjusting for sociodemographic variables, chronic conditions, health behaviours, and job characteristics. As a sensitivity analysis, we examined the associations between working hours and MDE in a sample also including those who were no longer employed at follow-up (total n = 2881) and adjusted for follow-up employment status (employed vs not). All analyses were performed using SAS 9.2 statistical software for Windows (Cary, Texas, USA).

## Results

### Characteristics of the baseline, follow-up and analytic samples

The three left columns of Table S1 present descriptive statistics for all the baseline participants (n = 7024) and the selected study samples (n = 6251 before and n = 2123 after exclusions of GHQ-30 cases, non-employed, and CIDI non-respondents). The participants in the analytic study sample were younger, more likely to be male, married or cohabiting, from higher occupational grades, less likely to have a chronic disease or to be a smoker and more likely to be in low strain or active jobs than the baseline sample including all baseline respondents. Some of these differences seemed to develop after the exclusion of GHQ-30 psychological morbidity cases which is as expected given that many of these characteristics are known correlates of psychological morbidity. Furthermore (not shown in the table), 54% of the original sample (vs. 52% in the study sample) worked standard 7–8 hours, 21% (vs. 21%) worked 9 hours, 15% (vs. 16%) worked 10 hours, and 10% (vs. 11%) worked 11–12 hours a day. Thus, any differences in working hours between the participants and the total baseline population were small.

As shown in Table S1 right-hand side columns, employees with long working hours were more likely to be men, married or cohabiting, from the higher occupational grades, those who had active jobs and high social support at work, and less often passive jobs or low strain jobs, than employees with standard working hours. They also tended to drink alcohol above the recommended limits, and were more likely to be ex-smokers and less likely to be never-smokers. Age and prevalence of chronic physical disease did not differ between categories of working hours.

### Predictors of major depressive episode

Sixty-six cases of MDE were identified at follow-up, resulting in a rate of 3.1% (Table 1). Predictors of the onset of depression were younger age, female sex, lower occupational grade, chronic physical disease, and moderate alcohol use (the odds ratio for depression for participants with alcohol use beyond the recommended limits was 2.19; in the expected direction but not statistically significant at conventional levels). We found no robust associations between marital status, smoking, job strain, or work social support and the onset of depression.

**Table 1:** Age- and sex-adjusted associations between baseline covariates and a major depressive episode at follow-up, the Whitehall II study, 1991–9.

Characteristic	N	N of cases	Odds ratio (95% CI)	*P-v*alue
Age			0.94 (0.89–0.99)	0.029
Sex				
Male	1626	41	1.00	
Female	497	25	2.08 (1.25–3.46)	0.005
Marital status				
Married/cohabiting	1717	49	1.00	
Non-married/-cohabiting	406	17	1.21 (0.68–2.16)	0.51
Occupational grade				
1 (highest)	385	4	1.00	
2	527	15	2.51 (0.82–7.67)	0.11
3	349	9	2.17 (0.66–7.15)	0.20
4	332	13	3.19 (1.02–9.99)	0.047
5	279	16	4.53 (1.47–13.90)	0.008
6 (lowest)	251	9	2.54 (0.74–8.69)	0.139
Chronic physical disease				
No	1454	33	1.00	
Yes	669	33	2.30 (1.41–3.78)	0.001
Alcohol use				
No	333	5	1.00	
Moderate	1432	51	2.68 (1.05–6.82)	0.038
High	358	10	2.19 (0.73–6.55)	0.16
Smoking				
Never	1105	28	1.00	
Ex	758	28	1.65 (0.96–2.83)	0.07
Current	260	10	1.46 (0.70–3.06)	0.31
Job strain				
Low strain	494	14	1.00	
Active	720	16	0.77 (0.37–1.59)	0.47
Passive	597	26	1.39 (0.71–2.71)	0.34
High strain	312	10	1.04 (0.46–2.39)	0.92
Social support at work				
High	699	21	1.00	
Intermediate	648	24	1.41 (0.77–2.56)	0.26
Low	776	21	1.11 (0.60–2.06)	0.73

CI = Confidence interval.

Working 11+ hours a day was related to a 2.43-fold odds of MDE compared to working 7 to 8 hours a day in an analysis adjusted for socio-demographic characteristics (Table 2). However, this effect was not seen until SES was introduced into the models (Model 3), suggesting that unadjusted associations are suppressed by SES. This is because high SES was related to an increased likelihood of working long hours (Table S1), but high SES is associated with a reduced risk of MDE (Table 1). Further adjustment for health and health behaviours made little change to the association (2.30). It was also robust to additional adjustment for work characteristics (job strain and social support at work), the odds ratio of MDE being 2.52-fold for 11+ working hours in the final model.

**Table 2:** Association between working hours at baseline and a major depressive episode at follow-up, the Whitehall II study, 1991-9.

Working Hours at Baseline	N	N of cases	Odds ratio (95% CI)*	*P-v*alue	Odds ratio (95% CI)†	*P-v*alue	Odds ratio (95% CI)‡	*P-v*alue	Odds ratio (95% CI)§	*P-v*alue	Odds ratio (95% CI)¶	*P-v*alue
7–8 hours	1105	38	1.00		1.00		1.00		1.00		1.00	
9 hours	445	8	0.51 (0.24–1.11)	0.09	0.57 (0.26–1.23)	0.15	0.68 (0.31–1.49)	0.33	0.62 (0.28–1.38)	0.24	0.66 (0.29–1.48)	0.31
10 hours	346	10	0.84 (0.41–1.70)	0.62	0.92 (0.45–1.88)	0.83	1.23 (0.59–2.58)	0.58	1.18 (0.56–2.51)	0.66	1.27 (0.59–2.72)	0.54
11–12 hours	227	10	1.29 (0.64–2.64)	0.48	1.55 (0.75–3.20)	0.24	2.43 (1.11–5.30)	0.026	2.30 (1.05–5.06)	0.038	2.52 (1.12–5.65)	0.025

*Unadjusted.

† Adjusted for age and sex.

‡ As previous model but additionally adjusted for occupational grade and marital status.

§ As previous model but additionally adjusted for chronic physical disease, smoking, and alcohol use.

¶ As previous model but additionally adjusted for job strain and social support at work.

CI = Confidence interval.

### Sensitivity analysis

As a sensitivity analysis, we examined the associations between working hours and MDE in a sample also including those who were no longer employed at follow-up (n = 2881). Working 11 hours or more at baseline was associated with a 1.89 odds (95% CI 0.92–3.86) of MDE at follow-up when compared to standard 7–8 hours. Further adjustment for employment status (employed vs. retired/other) made little change to the association (OR 1.87, 95% CI 0.91–3.84).

## Discussion

Working overtime predicted the onset of a major depressive episode in a middle-aged cohort of British civil servants, followed for an average of 5.8 years. Working 11 or more hours a day was associated with a 2.3- to 2.5-fold risk of an MDE when compared with working a standard 7–8 hours a day. This association was robust to adjustment for a range of socio-demographic, life-style, and work-related factors at baseline.

The main strengths of our study are its relatively large sample size, the prospective design and the use of the standardized CIDI interview which has been shown to be a valid measure of DSM-III-R non-psychotic disorders [15]. However, some limitations are noteworthy. First, the CIDI interview was only available at follow-up so baseline cases had to be excluded based on GHQ-30 caseness. However, the GHQ is a well-established scale for the evaluation of psychological morbidity in general population samples. In relation to diagnosed mental disorders, especially mood and anxiety disorders, the GHQ has shown good clinical validity [19]–[21]. As the GHQ-30 also detects a range of minor psychiatric disorders, such as subclinical depression, it is possible that our baseline exclusion of GHQ-30 cases is over zealous.

Second, being based on observational data, this investigation cannot rule out the possibility of residual confounding by other, unmeasured or imprecisely measured predictors of new-onset depression. We also found that the statistically significant association between 11+ weekly hours of work and the onset of depression was hidden in models not adjusted for individual SES. As SES was inversely associated with depression and positively associated with working hours, its effect can be considered as suppression, ‘a situation in which the magnitude of the relationship between an independent variable and a dependent variable becomes larger when a third variable is included’ [27]–[28]. In terms of prevention, revealing the relevance of long working hours as a risk factor among high-SES employees who otherwise have lower risk of depression seems important.

A further limitation relates to our inability to assess interaction effects due to the relatively small numbers of new-onset cases of MDE. For example, it is possible there are sex differences in the association between long working hours and mental health [8], [12]. Some positive work characteristics, such as high control or high rewards at work, may buffer an employee against the adverse health effects of long working hours [22], [29]–[35]. On the other hand, working long hours may also mean higher exposure to adverse working conditions. Further studies with larger sample sizes are needed to examine possible interaction effects in relation to clinical depression.

The rate of depression at follow-up was 3.1%. The median 1-year prevalence of depression across studies in general populations has been higher, approximately 5% [2]. This might be because only participants free of psychological distress at baseline were selected for follow-up. Furthermore, the Whitehall II study comprised a working population which has previously been shown to be healthier than the general population [36]. As all civil servants are white collar workers, it remains to be investigated whether our findings are generalizable to blue-collar workers and employees in the private sector.

Predictors of the onset of depression in our study were younger age, female sex, lower occupational grade, chronic physical disease, and moderate alcohol use (the odds ratio for depression in participants who used alcohol beyond the recommended limits was 2.19; in the expected direction but not statistically significant at conventional levels). We found no robust associations between marital status, smoking, job strain, or work social support and the onset of depression. Earlier literature suggests quite strong evidence for age (early or mid-adulthood), female sex, chronic physical disease such as CHD, binge drinking, smoking, low SES, and negative stressful life events as predictors of depression [35], [37]–[41]. Smoking intensity in white-collar employees, such as those in our study, is very likely to be lower than in the general population, which may be one explanation for the lack of an association with depression. Work stress and work social support have been suggested to be associated with depression [29]–[34], but a recent review focusing on clinical disorders [32] suggested that the association is inconclusive. However, in other analyses in this cohort we have found associations between job strain and depression, especially when the exposure to job strain was repeated [34].

Our sensitivity analysis of all follow-up participants irrespective of employment status revealed similar, although a weaker association between overtime work and depression compared to findings from analyses restricted to those employed at follow-up. These slightly weaker associations were expected given the possible misclassification of working hours during follow-up among those who were no longer employed. Some of those not employed may have stopped working several years before the onset of depression.

Our findings are in accordance with some observations from previous studies showing a positive association between long working hours and depression [12] but contrast with other reports of null findings [12]–[14]. Mixed findings in this field of research may relate to various operationalisations of long working hours, that is, in some studies the cut-point has been 40 [12] or 45 hours [14] or “more than one hour weekly overtime work” versus less [13] and possibly to the different work cultures in which these studies were carried out. Furthermore, some studies included part-time workers in the reference group although it is known that people with pre-existing health problems may choose part-time jobs [42]. For a better comparison between studies, a consistent definition for overtime and long working hours is needed in the future.

Plausible explanations of why long working hours are associated with the development of depression can not be drawn directly from our study. Serial adjustment for socio-demographic factors, physical disease, smoking, alcohol use, job strain, and social support at work, had little effect on the association or even strengthened it. Long working hours may in part affect mental health through factors not measured in our study, such as work-family conflicts [7], [43], [44], difficulties in unwinding after work [45] or prolonged increased cortisol levels [46]. The effect of long working hours on mental health may also be different in women and men [8], [12]. To date, the exact aetiology of depression is not known, but it is widely assumed that it is multifactorial involving genetic, biological, and psychosocial factors [35], [37], [38], [47].

In conclusion, this study suggests an association between long working hours and the onset of major depressive episode. Large-scale population-based cohort studies are needed to examine whether the association can be found in other contexts than Civil Service and intervention studies are needed to examine whether interventions designed to reduce working hours would alter depression risk in working populations.

## Supporting Information

Table S1 **Characteristics of the participants by daily working hours at baseline Data are N (%) or mean (S.D.), the Whitehall II study, 1991–9.**
(DOC)
