# Supplementary material for: Overtime Work as a Predictor of Major Depressive Episode: A 5-Year Follow-Up of the Whitehall II Study
Source: PLoS One. 2012 Jan 25;7(1):e30719. doi: 10.1371/journal.pone.0030719 (PMC3266289; doi:10.1371/journal.pone.0030719)
Supplement: Table S1 — Characteristics of the participants by daily working hours at baseline Data are N (%) or mean (S.D.), the Whitehall II study, 1991–9. (DOC) [file pone.0030719.s001.doc]

**Table S1. Characteristics of the participants by daily working hours at baseline Data are N (%) or mean (S.D.), the Whitehall II study, 1991-9**

|  | **Sample Selection** | | |  | **Working Hours Analytic sample N (%) / Mean (SD)** | | | | |
| --- | --- | --- | --- | --- | --- | --- | --- | --- | --- |
| **Characteristic** | **All baseline respondents (N=7024)** | **Follow-up respondents before exclusions* (N=6251)** | **Analytic study sample (N=2123)** |  | **7-8 hours (N=1105)** | **9 hours (N=445)** | **10 hours (N=346)** | **11-12 hours (N=227)** | ***P-v*alue†** |
| Age | 48.9 (5.7) | 48.8 (5.7) | 46.7 (4.8) |  | 46.6 (4.8) | 46.6 (4.6) | 47.2 (4.7) | 46.6 (4.8) | 0.23 |
| Sex |  |  |  |  |  |  |  |  | <0.0001 |
| Male | 4956 (71) | 4448 (71) | 1626 (77) |  | 781 (71) | 366 (82) | 274 (79) | 205 (90) |  |
| Female | 2068 (29) | 1803 (29) | 497 (23) |  | 324 (29) | 79 (18) | 72 (21) | 22 (10) |  |
| Marital status |  |  |  |  |  |  |  |  | <0.0001 |
| Married/cohabiting | 5393 (77) | 4826 (77) | 1717 (81) |  | 849 (77) | 366 (82) | 290 (84) | 212 (93) |  |
| Non-married/-cohabiting | 1631 (23) | 1425 (23) | 406 (19) |  | 256 (23) | 79 (18) | 56 (16) | 15 (7) |  |
| Occupational grade |  |  |  |  |  |  |  |  | <0.0001 |
| 1 (highest) | 1228 (17) | 1153 (18) | 385 (18) |  | 67 (6) | 103 (23) | 109 (32) | 106 (47) |  |
| 2 | 1543 (22) | 1403 (22) | 527 (25) |  | 216 (20) | 146 (33) | 110 (32) | 55 (24) |  |
| 3 | 995 (14) | 901 (14) | 349 (16) |  | 202 (18) | 79 (18) | 43 (12) | 25 (11) |  |
| 4 | 1205 (17) | 1070 (17) | 332 (16) |  | 224 (20) | 49 (11) | 34 (10) | 25 (11) |  |
| 5 | 959 (14) | 840 (13) | 279 (13) |  | 194 (18) | 47 (11) | 26 (8) | 12 (5) |  |
| 6 (lowest) | 1094 (16) | 884 (14) | 251 (12) |  | 202 (18) | 21 (5) | 24 (7) | 4 (2) |  |
| Chronic physical disease |  |  |  |  |  |  |  |  | 0.98 |
| No | 4468 (64) | 3977 (64) | 1454 (68) |  | 758 (69) | 301 (68) | 238 (69) | 157 (69) |  |
| Yes | 2556 (36) | 2274 (36) | 669 (32) |  | 347 (31) | 144 (32) | 108 (31) | 70 (31) |  |
| Alcohol use |  |  |  |  |  |  |  |  | 0.0004 |
| No | 1303 (19) | 1108 (18) | 333 (16) |  | 208 (19) | 57 (13) | 44 (13) | 24 (11) |  |
| Moderate | 4603 (66) | 4137 (66) | 1432 (67) |  | 732 (66) | 315 (71) | 233 (67) | 152 (67) |  |
| High | 1118 (16) | 1006 (16) | 358 (17) |  | 165 (15) | 73 (16) | 69 (20) | 51 (22) |  |
| Smoking |  |  |  |  |  |  |  |  | 0.003 |
| Never | 3585 (51) | 3227 (52) | 1105 (52) |  | 611 (55) | 230 (52) | 163 (47) | 101 (44) |  |
| Ex | 2446 (35) | 2189 (35) | 758 (36) |  | 352 (32) | 167 (38) | 137 (40) | 102 (45) |  |
| Current | 993 (14) | 835 (13) | 260 (12) |  | 142 (13) | 48 (11) | 46 (13) | 24 (11) |  |
| Job strain |  |  |  |  |  |  |  |  | <0.0001 |
| Low strain | 1354 (19) | 1231 (20) | 494 (23) |  | 279 (25) | 109 (24) | 76 (22) | 30 (13) |  |
| Active | 2185 (31) | 1976 (32) | 720 (34) |  | 199 (18) | 198 (44) | 177 (51) | 146 (64) |  |
| Passive | 2115 (30) | 1827 (29) | 597 (28) |  | 451 (41) | 74 (17) | 50 (14) | 22 (10) |  |
| High strain | 1370 (20) | 1217 (19) | 312 (15) |  | 176 (16) | 64 (14) | 43 (12) | 29 (13) |  |
| Social support at work |  |  |  |  |  |  |  |  | 0.019 |
| High | 2451 (35) | 2164 (35) | 699 (33) |  | 334 (30) | 163 (37) | 113 (33) | 89 (39) |  |
| Intermediate | 2010 (29) | 1807 (29) | 648 (31) |  | 336 (30) | 129 (29) | 109 (32) | 74 (33) |  |
| Low | 2563 (36) | 2280 (36) | 776 (37) |  | 435 (39) | 153 (34) | 124 (36) | 64 (28) |  |

*Before exclusion of GHQ-30 cases at baseline, non-employed at follow-up, and non-participants in CIDI interview at follow-up.

†*P* value for heterogeneity between the groups of working hours.
